# Mid-Liver Stage Arrest of *Plasmodium falciparum* Schizonts in Primary Porcine Hepatocytes

**DOI:** 10.3389/fcimb.2022.834850

**Published:** 2022-02-17

**Authors:** Saskia C. van der Boor, Geert-Jan van Gemert, Alex E. J. Hanssen, Youri M. van Waardenburg, Matthew B. B. McCall, Teun Bousema, Johannes H. W. de Wilt, Robert W. Sauerwein, Annie S. P. Yang

**Affiliations:** ^1^Radboudumc Center for Infectious Diseases, Department of Medical Microbiology, Radboud University Medical Center, Nijmegen, Netherlands; ^2^Animal Research Facility, Radboud University Medical Center, Nijmegen, Netherlands; ^3^Department of Surgery, Radboud University Medical Center, Nijmegen, Netherlands; ^4^TropIQ Health Sciences, Nijmegen, Netherlands

**Keywords:** malaria, hepatocyte, *Plasmodium falciparum*, schizont, porcine (pig) model

## Abstract

During co-evolution *Plasmodium* parasites and vertebrates went through a process of selection resulting in defined and preferred parasite-host combinations. As such, *Plasmodium falciparum (Pf*) sporozoites can infect human hepatocytes while seemingly incompatible with host cellular machinery of other species. The compatibility between parasite invasion ligands and their respective human hepatocyte receptors plays a key role in *Pf* host selectivity. However, it is unclear whether the ability of *Pf* sporozoites to mature in cross-species infection also plays a role in host tropism. Here we used fresh hepatocytes isolated from porcine livers to study permissiveness to *Pf* sporozoite invasion and development. We monitored intra-hepatic development *via* immunofluorescence using anti-HSP70, MSP1, EXP1, and EXP2 antibodies. Our data shows that *Pf* sporozoites can invade non-human hepatocytes and undergo partial maturation with a significant decrease in schizont numbers between day three and day five. A possible explanation is that *Pf* sporozoites fail to form a parasitophorous vacuolar membrane (PVM) during invasion. Indeed, the observed aberrant EXP1 and EXP2 staining supports the presence of an atypical PVM. Functions of the PVM include the transport of nutrients, export of waste, and offering a protective barrier against intracellular host effectors. Therefore, an atypical PVM likely results in deficiencies that may detrimentally impact parasite development at multiple levels. In summary, despite successful invasion of porcine hepatocytes, *Pf* development arrests at mid-stage, possibly due to an inability to mobilize critical nutrients across the PVM. These findings underscore the potential of a porcine liver model for understanding the importance of host factors required for *Pf* mid-liver stage development.

## Introduction

*Plasmodium falciparum* (*Pf*) is the causative agent for most of the mortality and morbidity associated with malaria. The disease begins when a human is bitten by an infectious mosquito and parasites (sporozoites) are injected into the skin and travel to the liver. In the liver, sporozoites traverse or transmigrate through multiple hepatocytes. Eventually, the parasites undergo productive invasion of a final hepatocyte, resulting in the parasite being surrounded by a parasitophorous vacuole made up of the parasitophorous vacuolar membrane (PVM) of host origin (Vaughan and Kappe, 2017). The PVM gets modified by the parasite as it develops and matures over the subsequent period of seven days, presumably to support the acquisition of host nutrients needed for parasite growth and to act as barrier against intracellular host defenses (Nyboer et al., 2018).

A comprehensive understanding of the key processes required for successful *Pf* invasion and maturation within the hepatocyte is lacking, including the molecular mechanisms that define host specificity (Mota et al., 2001; Mello-Vieira et al., 2020). Although key host receptors such as cluster of differentiation 81 (CD81) (Silvie et al., 2003), class B scavenger receptor type 1 (SR-B1) (Rodrigues et al., 2008), Ephrin A2 (Kaushansky et al., 2015), and highly sulfated heparin sulfate proteoglycans (HSPGs) (Coppi et al., 2007) have been identified as playing a role, their reciprocal interacting partners in the parasite remain largely unknown. Similarly, while there is an abundant number of parasite proteins implicated in sporozoite entry into hepatocytes such as circumsporozoite protein (CSP) [reviewed in (Sinnis and Coppi, 2007)], apical merozoite antigen 1 (AMA1), merozoite apical erythrocyte-binding ligand (MAEBL) (Yang et al., 2017), and the thrombospondin-related anonymous protein (TRAP) (Ejigiri et al., 2012), many of the corresponding host ligands remain to be identified. It is unclear why *Pf* sporozoites show exclusive human tropism, with natural infection appearing limited to humans (Liu et al., 2016; Sato, 2021), unlike their rodent malaria counterparts *P. yoelii* (*Py*) and *P. berghei* (*Pb*) (Prudencio et al., 2011). A prevailing theory is the narrow pre-defined window of parasite-host selectivity. For instance, Silvie et al. (2003) showed that *Pf* sporozoites are unable to achieve invasion in mouse hepatocytes while *Py* sporozoites can invade both human and mouse hepatocytes (Silvie et al., 2003). While this makes logical sense as mice are very evolutionary distant from humans, it remains unclear whether *Pf* can infect and mature in non-human hepatocytes from a more closely related species such as pigs, which have been used as general surgical models, as pharmacological models, in xenotransplantation research, in gene modification research, and in toxicology and immunological studies (Wernersson et al., 2005; Walters and Prather, 2013; Meier et al., 2015). Identifying potential hidden host reservoirs for malaria parasites would be critically relevant for elimination of the disease in the future. Additionally, a better understanding of the molecular mechanism behind sporozoite invasion and maturation is key to the identification of novel treatment targets. Here, we investigated whether *Pf* sporozoites can invade and mature in porcine hepatocytes.

## Materials and Methods

### Ethics Statement

Primary human hepatocytes were obtained as remnant surgical material from patients undergoing partial hepatectomy at the Radboud University Medical Center (Radboudumc). Human samples were anonymized, and general approval for use of remnant surgical material was granted in accordance to the Dutch ethical legislation, as described in the Medical Research (Human Subjects) Act and confirmed by the Committee on Research involving Human Subjects, in the region of Arnhem-Nijmegen, the Netherlands. Primary porcine hepatocytes were obtained as remnant surgical material from minipigs (*Sus scrofa domesticus*) undergoing surgical procedures for research and training purposes and were provided by the Animal Research Facility of the Radboudumc.

### Viability Assay

The viability of porcine hepatocytes was determined by measuring their overall mitochondrial enzymatic activity using 3-(4,5-dimethylthiazol-2-yl)-2,5-diphenyltetrazolium bromide (MTT). MTT was dissolved in phosphate buffered saline (PBS) at a concentration of 5 mg/mL and was added to each well. Hepatocytes were incubated for four hours at 37°C. Subsequently, the MTT solution was removed, and dimethyl sulfoxide (DMSO) was added to the culture for five minutes to dissolve formazan crystals. Next, the amount of converted MTT was determined by measuring the extinction of the well. The analysis was performed with the iMarkTM Microplate Reader (Bio-Rad). Extinction was measured at a wavelength of 570 nm. The reference wavelength was set at 630 nm.

### Generation of Sporozoites for *In Vitro* Infection

*Pf* NF135.C10 asexual stages were generated as described previously (Yang et al., 2021). This clone originates from a clinical isolate in Cambodia (Teirlinck et al., 2013). Briefly, asexual and sexual blood stages were cultured in a semi-automated culture system. Sporozoites were produced by feeding *Anopheles stephensi* (Sind-Kasur Nijmegen strain) using standard membrane feeding of cultured gametocytes. Salivary glands were dissected by hand, collected in William’s B medium [William’s E medium with Glutamax (Thermo Fisher, 32551-087), supplemented with 1× insulin/transferrin/selenium (Thermo Fisher, 41400-045), 1 mM sodium pyruvate (Thermo Fisher, 11360-070), 1× MEM-NEAA (Thermo Fisher, 11140-035), 2.5 µg/ml Fungizone (Thermo Fisher 15290-018), 200 U/ml penicillin/streptomycin (Thermo Fisher 15140-122), and 1.6 µM dexamethasone (Sigma-Aldrich D4902-100MG)], and homogenized in a custom-made glass grinder. Sporozoites were counted in a Bürker-Türk counting chamber using phase-contrast microscopy. All sporozoites were supplemented with heat inactivated human serum at 10% of the total volume prior to hepatocyte infection.

### Isolation and Infection of Primary Human and Porcine Hepatocytes

The isolation and infection of fresh hepatocytes has been described elsewhere (Walk et al., 2017; Yang et al., 2021). Briefly, viable hepatocytes were suspended in complete William’s B medium without serum. Hepatocytes were seeded into 96-well flat-bottom plates at a seeding density of 62.500 hepatocytes per well and cultured at 37°C in an atmosphere of 5% CO_2_ with daily media refreshment.

Two days after plating, sporozoites were added to the wells at a 1:1 ratio. Media was refreshed after three hours to remove non-invaded sporozoites and refreshed daily throughout the time-course. After time-course completion, cells were fixed with 4% paraformaldehyde (Thermo Fisher Scientific: catalogue number 28906) for ten minutes.

### Immunofluorescence Assay

After each incubation step, cells were washed thrice with PBS. First, fixed cells were permeabilized with 1% triton x-100 for ten minutes at room temperature. Subsequently, cells were incubated with 0.1 M glycine to avoid nonspecific binding of antibodies to free aldehyde groups. Cells were then incubated with 3% bovine serum albumin for forty-five minutes. Next, cells were incubated with primary antibodies for one hour at room temperature: rabbit anti-*Pf* HSP70 (Heat Shock Protein, 1:75 dilution), mouse anti-*Pf* MSP1 (Merozoites Surface Protein, 1:100 dilution), mouse anti-*Pf* EXP1 (Exported Protein 1, 1:1000 dilution), mouse anti-*Pf* EXP2 (Exported Protein 2, 1:1000 dilution) or goat anti-*Plasmodium berghei* (*Pb*) UIS4 (Upregulated in Infectious Sporozoites 4, 1:300 dilution). Cells were subsequently incubated with secondary antibodies (1:200 dilution) for one hour at room temperature in the dark: goat anti-rabbit Alexa 594 for *Pf*HSP70, donkey anti-mouse Alexa 647 for anti-*Pf*MSP1 staining, goat anti-mouse Alexa 488 for anti-*Pf*EXP1 and anti-*Pf*EXP2 staining, and donkey anti-goat Alexa 594. Subsequently, nuclei were stained with chromatin-specific 4’,6-diamidino-2-phenylindole (DAPI, 1:200 dilution) and incubated for one hour in the dark. Finally, 0.1% sodium azide was added. Cells were stored at 4°C.

### Microscopy

Monolayer viability was assessed by brightfield microscopy at 400x magnification. Following immunofluorescent staining, parasite invasion into hepatocytes was visualized with a Leica DMI6000B inverted epifluorescent high content microscope. For each well, a tile size of 9x9 was obtained using a 20x objective. Tiles were manually counted based on *Pf*HSP70 and DAPI positivity in FIJI. Schizonts were characterized using the region of interest (ROI) tool to determine the cross-sectional surface area, circumference, and raw integrated density (RawIntDen) of each manually encircled schizont. The RawIntDen values give the sum of all pixel values in the ROI. The RawIntDen value was corrected for background noise. Background noise was calculated by measuring the total intensity of a whole image divided by the area of the image. The final intensity of the parasite was calculated by subtracting the background value from the measured value. Confocal images were obtained with a Zeiss LSM880 microscope with Airyscan using a 63x (oil) objective and 2x zoom.

### Traversal Assay

A master mix was prepared including 10% heat inactivated human serum, 100 mg/ml FITC dextran, sporozoites (1:1 infection), and William’s B medium, and was added to each well. Controls included master mix containing no sporozoites, and master mix containing cytochalasin D (1:100 dilution). Cells were spun at 3000 revolutions per minute for ten minutes and stored at 37°C for two hours. Subsequently, cells were washed with PBS. Trypsin was added per well and cells were kept at 37°C for five minutes. Finally, 10% fetal bovine serum in PBS was added to each well. Traversal data was obtained by flow cytometry (Beckman Coulter Gallios 10-color), and analysis was performed using FlowJo software (version 10.0.8, Tree Star).

### Statistical Analysis

Three biological replicates were used for fresh porcine hepatocytes, and three biological replicates were used for human hepatocytes, of which two replicates were cryopreserved human hepatocytes and one was fresh. Each biological replicate had at least two technical replicates. All statistical tests were performed using Prism 9 (version 9.2.0, GraphPad Software, California USA).

## Results

### Traversal of Porcine Hepatocytes by *Plasmodium falciparum* Sporozoites

**Figure 1:**
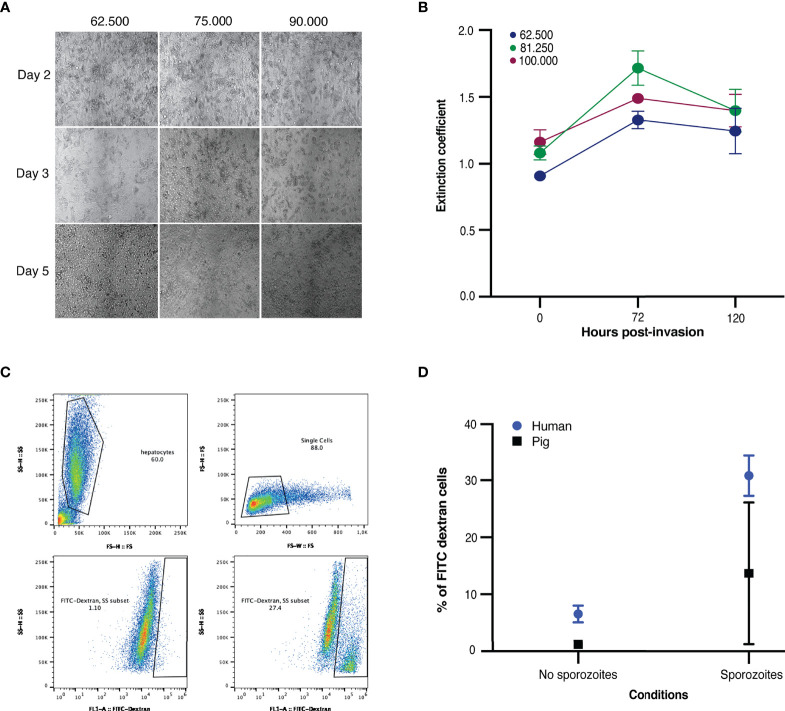
**(A)** Microscopic images of freshly seeded porcine hepatocytes at 62.500, 75.000 and 90.000 hepatocytes per well on days two, three and five post-invasion. **(B)** Viability of freshly isolated porcine hepatocytes. Porcine hepatocytes were seeded at densities of 62.500, 81.250 and 100.000 hepatocytes per well. MTT levels are depicted of uninfected control hepatocytes on day three, day five, and day seven post-invasion. Mean MTT reading and standard deviation of four technical replicates of two biological replicates. The extinction was measured at a wavelength of 570 nm. The reference wavelength was set at 630 nm. **(C, D)** Traversal of NF135.C10 sporozoites in fresh human and porcine hepatocytes three hours post-invasion of three biological donors, each with three technical replicates. **(C)** Fluorescence activated cell sorting gating strategy used to study traversal. Top left panel: selection of hepatocytes. Top right panel: selection of single cells. Bottom left panel: hepatocytes incubated in FITC-Dextran only (negative control). Bottom right panel: hepatocytes incubated with FITC-Dextran and sporozoites. For both bottom panels, the y-axis shows the degree of FITC-positivity in the cells. For all panels, each dot represents a cell/event measured. **(D)** Mean percentage (± SD) of traversed porcine and human hepatocytes.

Fresh porcine hepatocytes were isolated based on human protocols as described previously Yang et al. (2021) (Yang et al., 2021). Different seeding densities were compared, and the viability of each density was assessed using brightfield microscopy and mitochondrial enzymatic activity (MTT) from day of invasion up to day five (**Figures 1A, B****)**. For all seeding densities, metabolic activity increased post-plating (up to seventy-two hours post-invasion) and remained steady as measured by MTT (**Figure 1B**). After day seven, the viability of the culture decreased with clumping of cells observed by brightfield microscopy. An optimal seeding density of 62.500 was chosen, as at higher densities the host cells tended to clump instead of forming monolayers (image not shown). This is in line with the seeding density used for primary human hepatocytes (Yang et al., 2021).

Both human (freshly isolated and cryopreserved) and porcine (fresh) hepatocytes were subsequently infected with NF135.C10 sporozoites two days after plating. Traversal assays were performed for three hours post-invasion and a significant presence of FITC dextran-positive cells were detected, indicating NF135.C10 sporozoites were able to traverse porcine hepatocytes, albeit at a lower rate than in human hepatocytes. The percentage of traversed hepatocytes was lower in porcine donors compared to human donors (**Figures 1C, D****)**.

### *Plasmodium falciparum* Sporozoite Development in Primary Porcine Hepatocytes

**Figure 2:**
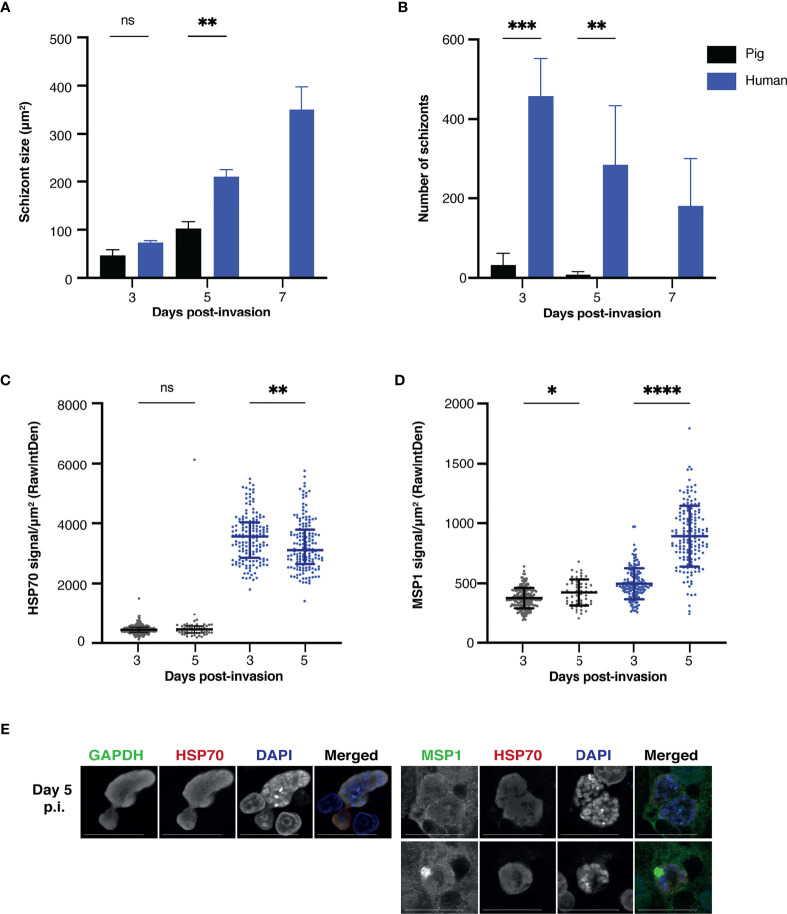
**(A)** Schizont size. Mean sizes (±SD) of NF135.C10 schizonts on day three, day five and day seven post-invasion in primary porcine and human hepatocytes based on immunofluorescence staining with HSP70. Per technical replicate, all or up to seventy-five schizonts were measured for each of the biological replicates (three human and three porcine donors). A two-way RM ANOVA was performed on the median of each replicate. Mean size (±SD) of NF135.C10 schizonts per technical replicate and circumference data is shown in **Supplementary Figure 1** and **Supplementary Table 1** **(B)** Schizont number. Mean number (±SD) of NF135.C10 schizonts on day three, day five and day seven post-invasion in primary porcine and human hepatocytes based on immunofluorescence staining with HSP70 and DAPI. All schizonts were counted per technical replicate of each of the biological replicates. A two-way RM ANOVA was performed on the median of each replicate. The mean number (±SD) of NF135.C10 schizonts per technical replicate is shown in **Supplementary Figure 2** **(C)** HSP70 signal. The median HSP70 signal (±IQR) measured per schizont on day three and day five post-invasion is shown, corrected for noise and schizont size. All or up to fifty schizonts were measured per technical replicate of the three biological replicates. A two-way RM ANOVA was performed on the median of each replicate. **(D)** MSP1 signal. Median MSP1 signal (±IQR) measured per schizont on day three and day five post-invasion, corrected for noise and schizont size. All or up to fifty schizonts were measured per technical replicate of three biological replicates. A two-way RM ANOVA was performed. **(E)** Expression of typical liver stage protein markers in porcine hepatocytes visualized by confocal microscopy including GAPDH, HSP70, MSP1 and DAPI. For comparative human images, please refer to (Yang et al., 2021). Objective 63×; zoom 2×; scale bar 25 µm. For panels A, C and D, the total number of schizonts analyzed per condition is shown in **Supplementary Table 2**. RawIntDen: raw integrated density. GAPDH, glyceraldehyde 3-phosphate dehydrogenase; HSP70, heat shock protein 70; IQR, interquartile range; MSP1, merozoite surface protein; NS, not significant; P.I., post-invasion; IQR, interquartile range. *P < 0.05; **P < 0.005; ***P < 0.001; ****P < 0.0001.

To assess whether infection of porcine hepatocytes was possible, liver cells from porcine and human donors were infected with NF135.C10 sporozoites. The number of intracellular schizonts was determined by immunofluorescence assay using DAPI and HSP70 staining on day three, five, and seven post-invasion (**Figure 2**). NF135.C10 schizonts of comparable size were detected in human and porcine hepatocytes on day three, illustrating the permissiveness of porcine hepatocytes to support *Pf* development (**Figure 2A**). The number of intracellular schizonts was significantly lower in porcine compared to human hepatocytes (**Figure 2B**) (p=0.0005 on day three and p=0.0074 on day five).

In the porcine cells, there was a significant decrease in schizont numbers from day three post-invasion onwards with no remaining schizonts left on day seven, indicating that parasites were unable to survive during this period. While a decrease in the number of intracellular schizonts was also observed in human hepatocytes, intracellular schizonts could still be detected on day seven post-invasion (**Figure 2B**). However, some degree of parasite nuclear division was observed in schizonts developing in porcine host hepatocytes, as illustrated by DAPI staining (**Figure 2E**). The integrated HSP70 signal intensity per schizont surface area remained relatively constant between day three and five post-invasion for both human and porcine hepatocytes, suggesting that the health of these parasites remained stable (**Figure 2C**). As expected, the signal was higher for parasites developing in the human hepatocytes as it is the ideal host for the parasite. Furthermore, a large increase in the MSP1 signal intensity per schizont size, a marker of maturation, was only observed in schizonts present in human hepatocytes (**Figure 2D**), indicating the ability of these schizonts to mature in the human host but not in the porcine host. Finally, parasites developing in the porcine host displayed typical liver stage markers such as HSP70, GAPDH and MSP1 (**Figure 2E**).

While schizonts developing in the porcine and human hepatocytes did not differ in size on day three post-invasion, there was a significant difference on day five post-invasion (p=0.0038) (**Figure 2A**). Schizonts developing in human hepatocytes increased in size from day three to day five (p=0.0008) and from day five to day seven (p=0.0006). In contrast, no significant difference in size was found from day three to day five in surviving schizonts in porcine hepatocytes (p=0.1137), indicating again that maturation is hindered in non-human host cells. Circumference data is shown in supplementary material and showed a similar trend (**Figure S1****)**.

### Developing Schizonts Show Aberrant EXP1 and EXP2 Expression

**Figure 3:**
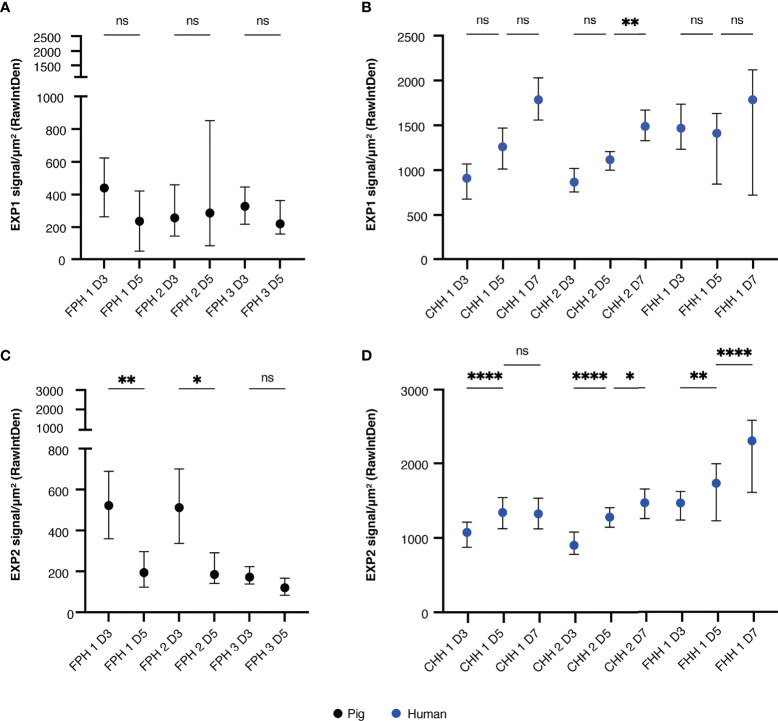
Median EXP signal (±IQR) measured per schizont in porcine hepatocytes on day three and day five post-invasion, corrected for noise and schizont size. All or up to seventy-five schizonts were measured per technical replicate of three biological replicates. A two-way RM ANOVA was performed on the median of each replicate. **(A)** EXP1 signal in porcine hepatocytes. **(B)** EXP1 signal in human hepatocytes. **(C)** EXP2 signal in porcine hepatocytes. **(D)** EXP2 signal in human hepatocytes. For all panels, the total number of schizonts analyzed per condition is shown in **Supplementary Table 2**. CHH, cryopreserved human hepatocytes; EXP, exported protein; FHH, fresh human hepatocytes; FPH, fresh porcine hepatocytes; NS, not significant. *P < 0.05; **P < 0.005; ****P < 0.0001.

As shown by the disappearance of schizonts from day five to day seven post-invasion as well as the steady MSP1 signal, it appears that schizonts cannot mature properly in the porcine hepatocyte system. The integrity of the PVM was next assessed using antibodies against two established PVM proteins, EXP1 and 2 (van Dijk et al., 2005; Labaied et al., 2007; Ploemen et al., 2012; Annoura et al., 2014; Kalanon et al., 2016; Manzoni et al., 2017; Yang et al., 2021). EXP1 and EXP2 have been identified as crucial PVM components for nutrient uptake and protein export in blood stages while a defined role in liver stage development remains elusive (Charnaud et al., 2018; Garten et al., 2018; Mesén-Ramírez et al., 2019; Nessel et al., 2020). In porcine hepatocytes, there was no apparent increase in EXP1 signal (**Figure 3A**). Although not significant, there was an increasing trend in EXP1 signal intensity observed from day three to day five for schizonts developing in human hepatocytes for some donors (**Figure 3B**). With regards to the EXP2 signal, there was a decrease in signal in schizonts developing in porcine hepatocytes from day three to day five whereas an increase was observed over time for schizonts in human hepatocytes (**Figures 3C, D****)**.

**Figure 4:**
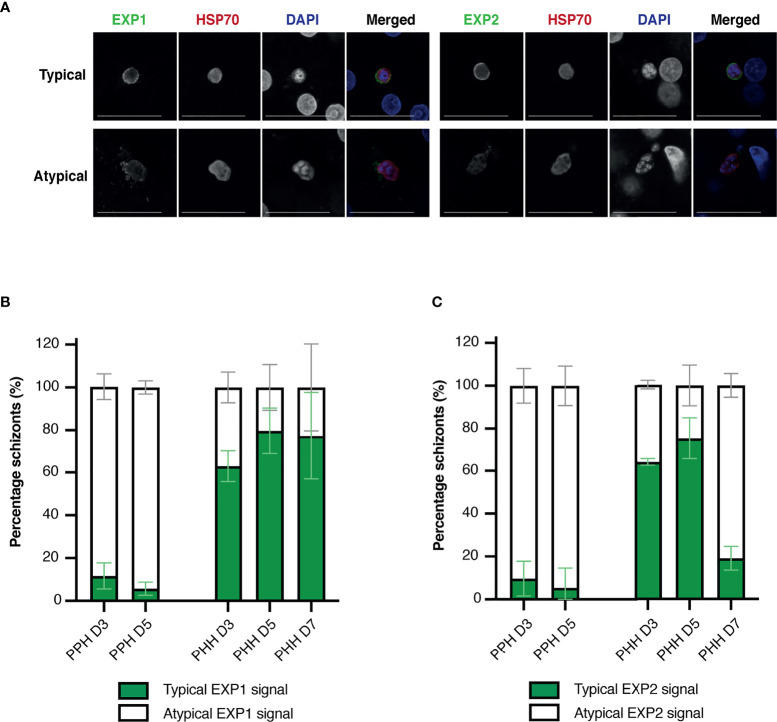
**(A)** Representative immunofluorescence images of typical liver stage protein markers visualized through confocal microscopy including EXP1 and EXP2, HSP70, and DAPI, classified as a typical (top) or atypical (bottom) staining pattern. Images shown were obtained from infected porcine hepatocytes. Objective 63×; zoom 2×; scale bar 25 µm. **(B, C)** Mean percentage of human and porcine schizonts with a typical or atypical EXP1(B) or EXP2 **(C)** staining pattern. Porcine hepatocytes are depicted on the left and human hepatocytes on the right of each bar plot. All or up to seventy-five schizonts were measured per technical replicate of three biological replicates. Results per biological replicate are shown in **Supplementary Figure 4**. For panels B and C, the total number of schizonts analyzed per condition is shown in **Supplementary Table 2**. EXP, exported protein; HSP, heat shock protein; PHH, primary human hepatocytes; PPH, primary porcine hepatocytes.

Two main staining patterns of EXP1 and EXP2 were observed and classified as typical (circle surrounding HSP70 and DAPI staining) and atypical (**Figure 4A** and **Figure S3A**). The majority of schizonts developing in porcine hepatocytes showed atypical EXP1 and EXP2 staining, compared to schizonts developing in human hepatocytes that showed mostly a typical EXP1 and EXP2 staining pattern (**Figures 4B, C****)**. Schizonts developing in porcine hepatocytes showed an increasing trend of atypical EXP1 and EXP2 staining over time, whereas the proportion of schizonts developing in human hepatocytes with a typical pattern increased over time, although this difference was not significant.

Additionally, fresh porcine hepatocytes were infected with the rodent *Pb*, showing that UIS4 localized to the PVM and is expressed throughout liver stage development in *Pb* (Mueller et al., 2005). Unlike porcine hepatocytes infected with *Pf, Pb* schizonts developing in porcine hepatocytes all showed a typical staining pattern for UIS4 (**Figure S3B**), suggestive for an intact PVM. Finally, schizonts developing in porcine hepatocytes with atypical EXP1 and EXP2 signal did not show a difference in schizont area (determined by measuring HSP70 signal) compared to schizonts with a typical EXP1 and EXP2 signal (**Figure S4****)**, whereas in human hepatocytes schizonts with a typical pattern appeared to be non-significantly larger.

## Discussion

Here, we show that *Pf* is capable of traversing, invading and partially maturing in non-human porcine hepatocytes. Incomplete maturation of schizonts may be due to an aberrant PVM, supported by the increasingly atypical EXP1 and EXP2 expression pattern observed over time. Additionally, the aberrant EXP1 and EXP2 staining suggest that parasite nutrient uptake may be impaired, which could hamper further maturation of the parasites.

*Pf* shows a remarkably narrow host range compared to other *Plasmodium* species and appears to uniquely infect primates *in vivo* and *in vitro* (Collins et al., 1994; Rathore et al., 2003). This selectivity is partially explained by different invasion requirements between *Plasmodium* species, as well as incompatibility of invasion proteins between *Pf* and the non-human host hepatocyte (i.e. a lock and key model), including CSP and CD81 (Rathore et al., 2003; Silvie et al., 2003; Silvie et al., 2006). As *Pf* is successful in invading porcine hepatocytes, albeit at a lower rate, it suggests that compatibility of parasite invasion ligands with their host counterparts is not the only requirement needed for parasite survival within a hepatocyte.

A key finding is the abrupt mid-liver stage arrest in parasite development coinciding with the increasingly aberrant EXP1 and EXP2 expression, established markers for PVM quality. The PVM forms a barrier with the host environment and is extensively modified by the parasite to provide the appropriate machinery to scavenge sufficient host nutrients and export proteins, whilst simultaneously offering protection from the hepatocytes’ defense mechanisms (Spielmann et al., 2012). Studies with *Pb* and *Py* show that complete cytosolic development of schizonts lacking an intact PVM is possible but generally schizonts only develop up to early-liver stage at twenty-four hours post-invasion (van Dijk et al., 2005; Silvie et al., 2006; Ploemen et al., 2012). Sporozoites lacking SLARP/SAP1 (sporozoite Asparagine-rich protein) arrest early (twenty-four hours post-invasion) and fail to express amongst others effector proteins P52, UIS4 and EXP1 (Aly et al., 2008). These proteins are critical for the formation of a PVM and lead to near-complete developmental arrest during early development (Aly et al., 2008). Sporozoites with P52/p36 deletions are incapable of productively invading hepatocytes resulting in near-complete attenuation during early liver stage in *Pb* and *Py*, as well as *Pf* (Ishino et al., 2005; van Dijk et al., 2005; Labaied et al., 2007; VanBuskirk et al., 2009; Ploemen et al., 2012; Manzoni et al., 2017). Finally, the function of B9 remains more controversial in different *Plasmodia* species: while required in *Pb* for productive hepatocyte invasion, this is not the case for *Pf* invasion into human hepatocytes despite structural similarity (Annoura et al., 2014; Fernandes et al., 2021). As schizonts are detected in porcine hepatocytes on day three and (to a lesser amount) on day five, this challenges this prevailing theory that a functional, intact, PVM is required for progression beyond early-stage *Pf* infection.

An important limitation is that we do not understand the mechanism behind the atypical PVM in porcine hepatocytes. This abnormal event could be due to several factors or a combination thereof, including refractory porcine hepatocytes for productive invasion, the inability of parasites to maintain the PVM, as well as host factors that may compromise the integrity of the PVM. We find that sporozoites do show characteristics of early transformation into normal liver-stage schizonts, as illustrated by the increase in nuclear material and presence of nuclear division. However, the increasingly aberrant expression of EXP1 and EXP2 supports the failure of schizonts to complete maturation up to day seven. The lack of fluorescently tagged EXP1/2 prevented live-imaging and we therefore cannot exactly pinpoint the timing of the loss of EXP1/2 from the PVM over time. This loss may be due to deteriorating parasite health or active degradation by the host. Alternatively, aberrant EXP1 and EXP2 expression of sporozoites in porcine hepatocytes could affect proper parasite development due impaired nutrient scavenging from the host environment.

Mello-Vieira et al. (2020) show that EXP2 is required for liver invasion and maturation and for establishing blood stage infection in a mouse model, which corroborates with our findings of its importance in maturation (Mello-Vieira et al., 2020). Furthermore, the EXP1 C-terminal region exposed to the host cytoplasm interacts with host Apolipoprotein A (ApoH) which is pivotal for successful liver-stage development in *Pb*, although it is unclear whether it performs the same role in *Pf* (Sá et al., 2017; Mesén-Ramírez et al., 2019; Wolanin et al., 2019). EXP1 is continuously trafficking to the PVM throughout the first days of the liver stage (Sá et al., 2017). As the parasite matures, its survival becomes increasingly dependent on its ability to scavenge lipids and nutrients from its host cell, for which it requires a functional PVM (Agop-Nersesian et al., 2018). The combined findings in rodent models may be a potential explanation for the inability of *Pf* to fully mature in porcine hepatocytes.

An aberrant PVM additionally exposes the developing parasites to the hostile host environment and host cell defense mechanisms. Although host responses to the invading parasites may vary between parasite species, targeting intracellular parasites is a conserved defense strategy of infected hepatocytes (Wacker et al., 2017). During parasite infection, diverse host autophagy pathways can be activated in hepatocytes (reviewed in (Agop-Nersesian et al., 2018)). This includes marking the PVM with the autophagy marker LC3, which activates the host cell autophagy machinery to eliminate intracellular parasites. The observed aberrant PVM may be the result of active host destruction of the PVM: the parasite proteins involved in PVM maintenance in human hepatocytes may be incompatible to those in porcine hepatocytes. This could impact the parasites’ ability to secure host resources and protect itself against host degradation.

To our knowledge, *Pf* infection of porcine hepatocytes has not been reported before. Our findings are confined to an *in vitro* system and it is not clear whether a similar mechanism of invasion and maturation occurs *in vivo*. Additionally, the cause of an impaired PVM remains unknown. The aberrant PVM could make the parasite susceptible to or may be a direct response to intracellular host defence mechanisms. Furthermore, these results underline the importance of EXP1 and EXP2 in *Pf* liver stage development and maturation, although their exact roles are not explored here. Future studies with *Pf*EXP-1 and EXP-2 knockdown parasites, as well as other PVM proteins, may be able to delineate the key processes involved in parasite maturation.

Altogether, our findings highlight the importance of understanding species-specific intracellular host factors involved in *Pf* liver-stage development. The partial development of *Pf* sporozoites in porcine hepatocytes underscores its potential as a platform to study early to mid-stage *Pf* liver-stage development, and to identify essential *Pf* and host proteins required for the formation and maintenance of the PVM. Additionally, the model may provide a novel platform to study host factors and nutrients required for full *Pf* liver-stage development.

## Data Availability Statement

The raw data supporting the conclusions of this article will be made available by the authors when possible and upon request

## Author Contributions

The study was conceptualized and designed by SB, AY, and RS. SB, AY, YW, GJG, and AH performed the experiments. JW coordinated the collection of fresh human liver segments. SB, AY, RS, TB, and MM were involved in conceptualization of the data. SC and AY analyzed the data and made figures for the article. SB and AY wrote the first manuscript. All authors contributed and reviewed the manuscript and approved the submitted version.

## Funding

This study was funded by the Department of Medical Microbiology, Radboud University Medical Center. AY is funded by the Dutch Research Council (NWO) talent scheme veni (VI.Veni.192.171).

## Conflict of Interest

Author RS was employed by company TropIQ Health Sciences.

The remaining authors declare that the research was conducted in the absence of any commercial or financial relationships that could be construed as a potential conflict of interest.

## Publisher’s Note

All claims expressed in this article are solely those of the authors and do not necessarily represent those of their affiliated organizations, or those of the publisher, the editors and the reviewers. Any product that may be evaluated in this article, or claim that may be made by its manufacturer, is not guaranteed or endorsed by the publisher.

## References

[B1] Agop-Nersesian C. Niklaus L. Wacker R. Theo Heussler V. (2018). Host Cell Cytosolic Immune Response During Plasmodium Liver Stage Development. FEMS Microbiol. Rev. 42, 3. doi: 10.1093/femsre/fuy007 PMC5995216 29529207

[B2] Aly A. S. Mikolajczak S. A. Rivera H. S. Camargo N. Jacobs-Lorena V. Labaied M.. (2008). Targeted Deletion of SAP1 Abolishes the Expression of Infectivity Factors Necessary for Successful Malaria Parasite Liver Infection. Mol. Microbiol. 69, 1. doi: 10.1111/j.1365-2958.2008.06271.x 18466298 PMC2615191

[B3] Annoura T. van Schaijk B. C. Ploemen I. H. Sajid M. Lin J. W. Vos M. W.. (2014). Two Plasmodium 6-Cys Family-Related Proteins Have Distinct and Critical Roles in Liver-Stage Development. FASEB J. 28, 5. doi: 10.1096/fj.13-241570 24509910

[B4] Charnaud S. C. Jonsdottir T. K. Ploemen P. R. Sajid H. E. Lin B. K. Vos B.. (2018). Spatial Organization of Protein Export in Malaria Parasite Blood Stages. Traffic 19, 8. doi: 10.1111/tra.12577 29696751

[B5] Collins W. E. Galland G. G. Sullivan J. S. Morris C. L. (1994). Selection of Different Strains of Plasmodium Falciparum for Testing Blood-Stage Vaccines in Aotus Nancymai Monkeys. Am. J. Trop. Med. Hyg 51, 2. doi: 10.4269/ajtmh.1994.51.224 8074257

[B6] Coppi A. Tewari R. Bishop J. R. Bennett B. L. Lawrence R. Esko J. D.. (2007). Heparan Sulfate Proteoglycans Provide a Signal to Plasmodium Sporozoites to Stop Migrating and Productively Invade Host Cells. Cell. Host. Microbe 2, 5. doi: 10.1016/j.chom.2007.10.002 18005753 PMC2117360

[B7] Ejigiri I. Ragheb D. R. Pino P. Coppi A. Bennett B. L. Soldati-Favre D.. (2012). Shedding of TRAP by a Rhomboid Protease From the Malaria Sporozoite Surface is Essential for Gliding Motility and Sporozoite Infectivity. PloS Path. 8, 7. doi: 10.1371/journal.ppat.1002725 PMC3406075 22911675

[B8] Fernandes P. Loubens M. Marinach C. Coppée R. Grand M. Andre T. -P.. (2021). Plasmodium Sporozoites Require the Protein B9 to Invade Hepatocytes. bioRxiv. doi: 10.1101/2021.10.25.465731 PMC9906020 36761022

[B9] Garten M. Nasamu A. S. Niles J. C. Zimmerberg J. Goldberg D. E. Beck J. (2018). EXP2 Is a Nutrient-Permeable Channel in the Vacuolar Membrane of Plasmodium and is Essential for Protein Export *via* PTEX. Nat. Microbiol. 3, 10. doi: 10.1038/s41564-018-0222-7 PMC6158082 30150733

[B10] Ishino T. Chinzei Y. Yuda M. (2005). Two Proteins With 6-Cys Motifs are Required for Malarial Parasites to Commit to Infection of the Hepatocyte. Mol. Microbiol. 58, 5, 1264–1275. doi: 10.1111/j.1365-2958.2005.04801.x 16313615

[B11] Kalanon M. Bargieri D. Sturm A. Matthews K. Ghosh S. Goodman C. D.. (2016). The Plasmodium Translocon of Exported Proteins Component EXP2 is Critical for Establishing a Patent Malaria Infection in Mice. Cell. Microbiol. 18 (3), 399–412. doi: 10.1111/cmi.12520 26347246

[B12] Kaushansky A. Douglass A. N. Arang N. Vigdorovich V. Dambrauskas N. Kain H. S.. (2015). Malaria Parasites Target the Hepatocyte Receptor Epha2 for Successful Host Infection. Science 350 (6264), 1089–1092. doi: 10.1126/science.aad3318 26612952 PMC4783171

[B13] Labaied M. Harupa A. Dumpit R. F. Coppens I. Mikolajczak S. A. Kappe S. H.. (2007). Plasmodium Yoelii Sporozoites With Simultaneous Deletion of P52 and P36 are Completely Attenuated and Confer Sterile Immunity Against Infection. Infect. Immun. 75 (8), 3758–3768. doi: 10.1128/iai.00225-07 17517871 PMC1951999

[B14] Liu W. Sundararaman S. A. Loy D. E. Learn G. H. Li Y. Plenderleith L. J.. (2016). Multigenomic Delineation of Plasmodium Species of the Laverania Subgenus Infecting Wild-Living Chimpanzees and Gorillas. Genome Biol. Evol. 8 (6), 1929–1939. doi: 10.1093/gbe/evw128 27289102 PMC4943199

[B15] Manzoni G. Marinach C. Topçu S. Briquet S. Grand M. Tolle M.. (2017). Plasmodium P36 Determines Host Cell Receptor Usage During Sporozoite Invasion. eLife 6, e25903. doi: 10.7554/eLife.25903 28506360 PMC5470872

[B16] Meier R. P. H. Navarro-Alvarez N. Morel P. Schuurman H. J. Strom S. Bühler L. H.. (2015). Current Status of Hepatocyte Xenotransplantation. Int. J. Surg. 23 (Pt B), 273–279. doi: 10.1016/j.ijsu.2015.08.077 26361861

[B17] Mello-Vieira J. Enguita F. J. de Koning-Ward T. F. Zuzarte-Luís V. Mota M. M.. (2020). Plasmodium Translocon Component EXP2 Facilitates Hepatocyte Invasion. Nat. Commun. 11, 1. doi: 10.1038/s41467-020-19492-4 33159090 PMC7648069

[B18] Mesén-Ramírez P. Bergmann B. Tran T. T. Garten M. Stäcker J. Naranjo-Prado I.. (2019). EXP1 is Critical for Nutrient Uptake Across the Parasitophorous Vacuole Membrane of Malaria Parasites. PloS Biol. 17, 9. doi: 10.1371/journal.pbio.3000473 PMC6786648 31568532

[B19] Mota M. M. Pradel G. Vanderberg J. P. Hafalla J. C. Frevert U. Nussenzweig R. S.. (2001). Migration of Plasmodium Sporozoites Through Cells Before Infection. Science 291 (5501), 141–144 . doi: 10.1126/science.291.5501.141 11141568

[B20] Mueller A. K. Camargo N. Kaiser K. Andorfer C. Frevert U. Matuschewski K.. (2005). Plasmodium Liver Stage Developmental Arrest by Depletion of a Protein at the Parasite-Host Interface. Proc. Natl. Acad. Sci. U. S. A. 102, 8. doi: 10.1073/pnas.0408442102 15699336 PMC548321

[B21] Nessel T. Beck J. M. Rayatpisheh S. Jami-Alahmadi Y. Wohlschlegel J. A. Goldberg D. E.. (2020). EXP1 is Required for Organisation of EXP2 in the Intraerythrocytic Malaria Parasite Vacuole. Cell Microbiol. 22, 5. doi: 10.1111/cmi.13168 PMC7138706 31990132

[B22] Nyboer B. Heiss K. Mueller A. K. Ingmundson A. (2018). The Plasmodium Liver-Stage Parasitophorous Vacuole: A Front-Line of Communication Between Parasite and Host. Int. J. Med. Microbiol. 308, 1. doi: 10.1016/j.ijmm.2017.09.008 28964681

[B23] Ploemen I. H. Croes H. J. van Gemert G. J. Wijers-Rouw M. Hermsen C. C. Sauerwein R. W.. (2012). Plasmodium Berghei Δp52&P36 Parasites Develop Independent of a Parasitophorous Vacuole Membrane in Huh-7 Liver Cells. PloS One 7, 12. doi: 10.1371/journal.pone.0050772 PMC3515443 23227206

[B24] Prudencio M. Mota M. M. Mendes A. M. (2011). A Toolbox to Study Liver Stage Malaria. Trends Parasitol. 27, 12. doi: 10.1016/j.pt.2011.09.004 22015112

[B25] Rathore D. Hrstka S. C. Sacci J. B. Jr De la Vega P. Linhardt R. J. Kumar S.. (2003). Molecular Mechanism of Host Specificity in Plasmodium Falciparum Infection: Role of Circumsporozoite Protein. J. Biol. Chem. 278, 42. doi: 10.1074/jbc.M306250200 12904297

[B26] Rodrigues C. D. Hannus M. Prudêncio M. Martin C. Gonçalves L. A. Portugal S.. (2008). Host Scavenger Receptor SR-BI Plays a Dual Role in the Establishment of Malaria Parasite Liver Infection. Cell. Host. Microbe 4, 3. doi: 10.1016/j.chom.2008.07.012 18779053

[B27] Sá E. Cunha C. Nyboer B. Heiss K. Sanches-Vaz M. Fontinha D.. (2017). Plasmodium Berghei EXP-1 Interacts With Host Apolipoprotein H During Plasmodium Liver-Stage Development. Proc. Natl. Acad. Sci. U. S. A. 114, 7. doi: 10.1073/pnas.1606419114 28137845 PMC5320984

[B28] Sato S. (2021). Plasmodium—A Brief Introduction to the Parasites Causing Human Malaria and Their Basic Biology. J. Physiol. Anthropol. 40, 1. doi: 10.1186/s40101-020-00251-9 33413683 PMC7792015

[B29] Silvie O. Greco C. Franetich J. F. Dubart-Kupperschmitt A. Hannoun L. van Gemert G. J.. (2003). Hepatocyte CD81 is Required for Plasmodium Falciparum and Plasmodium Yoelii Sporozoite Infectivity. Nat. Med. 9, 1. doi: 10.1038/nm808 12483205

[B30] Silvie O. Rubinstein E. Franetich J. F. Prenant M. Belnoue E. Rénia L.. (2006). Expression of Human CD81 Differently Affects Host Cell Susceptibility to Malaria Sporozoites Depending on the Plasmodium Species. Cell. Microbiol. 8, 7. doi: 10.1111/j.1462-5822.2006.00697.x 16819966

[B31] Sinnis P. Coppi A. (2007). A Long and Winding Road: The Plasmodium Sporozoite’s Journey in the Mammalian Host. Parasitol. Int. 56, 3. doi: 10.1016/j.parint.2007.04.002 17513164 PMC1995443

[B32] Spielmann T. Montagna G. N. Hecht L. Matuschewski K. (2012). Molecular Make-Up of the Plasmodium Parasitophorous Vacuolar Membrane. Int. J. Med. Microbiol. 302, 4–5. doi: 10.1016/j.ijmm.2012.07.011 22898489

[B33] Teirlinck A. C. Roestenberg M. van de Vegte-Bolmer M. Scholzen A. Heinrichs M. J. Siebelink-Stoter R.. (2013). NF135.C10: A New Plasmodium Falciparum Clone for Controlled Human Malaria Infections. J. Infect. Dis. 207 (4), 656–660. doi: 10.1093/infdis/jis725 23186785 PMC3549599

[B34] VanBuskirk K. M. O'Neill M. T. De La Vega P. Maier A. G. Krzych U. Williams J.. (2009). Preerythrocytic, Live-Attenuated Plasmodium Falciparum Vaccine Candidates by Design. Proc. Natl. Acad. Sci. U. S. A. 106, 31. doi: 10.1073/pnas.0906387106 PMC2714279 19625622

[B35] van Dijk M. R. Douradinha B. Franke-Fayard B. Heussler V. van Dooren M.W. van Schaijk B.. (2005). Genetically Attenuated, P36p-Deficient Malarial Sporozoites Induce Protective Immunity and Apoptosis of Infected Liver Cells. Proc. Natl. Acad. Sci. U. S. A. 102, 34. doi: 10.1073/pnas.0500925102 16103357 PMC1189305

[B36] Vaughan A. M. Kappe S. H. I. (2017). Malaria Parasite Liver Infection and Exoerythrocytic Biology. Cold Spring Harb. Perspect. Med. 7, 6. doi: 10.1101/cshperspect.a025486 PMC5453383 28242785

[B37] Wacker R. Eickel N. Schmuckli-Maurer J. Annoura T. Niklaus L. Khan S. M.. (2017). LC3-Association With the Parasitophorous Vacuole Membrane of Plasmodium Berghei Liver Stages Follows a Noncanonical Autophagy Pathway. Cell. Microbiol. 19, 10. doi: 10.1111/cmi.12754 28573684

[B38] Walk J. Reuling I. J. Behet M. C. Meerstein-Kessel L. Graumans W. van Gemert G. J.. (2017). Modest Heterologous Protection After Plasmodium Falciparum Sporozoite Immunization: A Double-Blind Randomized Controlled Clinical Trial. BMC Med. 15 (1), 168. doi: 10.1186/s12916-017-0923-4 28903777 PMC5598044

[B39] Walters E. M. Prather R. S. (2013). Advancing Swine Models for Human Health and Diseases. Mo. Med. 110 (3), 212–215. 23829105 PMC6179855

[B40] Wernersson R. Schierup M. H. Jørgensen F. G. Gorodkin J. Panitz F. Staerfeldt H. H.. (2005). Pigs in Sequence Space: A 0.66X Coverage Pig Genome Survey Based on Shotgun Sequencing. BMC Genomics 6, 70. doi: 10.1186/1471-2164-6-70 15885146 PMC1142312

[B41] Wolanin K. Fontinha D. Sanches-Vaz M. Nyboer M. Heiss B. Mueller K.. (2019). A Crucial Role for the C-Terminal Domain of Exported Protein 1 During the Mosquito and Hepatic Stages of the Plasmodium Berghei Life Cycle. Cell Microbiol. 21 (10), e13088. doi: 10.1111/cmi.13088 31364224 PMC6771729

[B42] Yang A. S. P. Lopaticki S. O'Neill M. T. Erickson S. M. Douglas D. N. Kneteman N. M.. (2017). AMA1 and MAEBL are Important for Plasmodium Falciparum Sporozoite Infection of the Liver. Cell. Microbiol. 19, (9). doi: 10.1111/cmi.12745 28371168

[B43] Yang A. S. P. van Waardenburg Y. M. van de Vegte-Bolmer M. van Gemert G. A. Graumans W. Wilt J. H. W.. (2021). Zonal Human Hepatocytes are Differentially Permissive to Plasmodium Falciparum Malaria Parasites. EMBO J. 40 (6), e106583. doi: 10.15252/embj.2020106583 33459428 PMC7957391

