# Supplementary material for: Mid-Liver Stage Arrest of Plasmodium falciparum Schizonts in Primary Porcine Hepatocytes
Source: Front Cell Infect Microbiol. 2022 Feb 17;12:834850. doi: 10.3389/fcimb.2022.834850 (PMC8892583; doi:10.3389/fcimb.2022.834850)
Supplement: Supplementary file 1 [file DataSheet_1.docx]

Supplementary Material

# Supplementary Figures and Tables

## Supplementary Figures


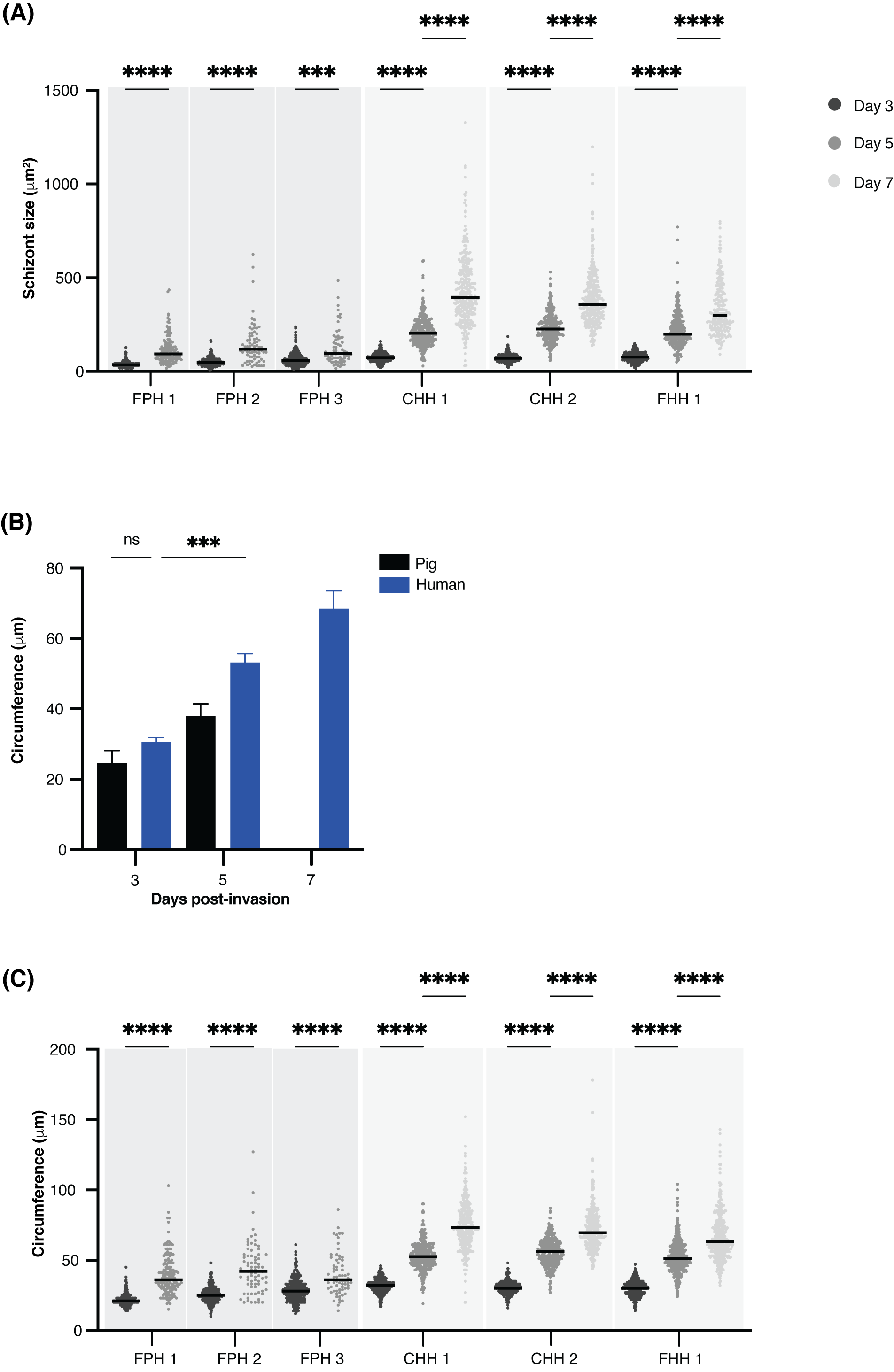


**Supplementary figure 1. (A)** Median NF135.C10 schizont size on day three, day five, and day seven, developing in primary porcine and human hepatocytes, as determined by immunofluorescence staining with HSP70. Data is shown per donor. Per technical replicate, all or up to seventy-five schizonts were measured. A two-way RM ANOVA was performed. **(B)** Mean circumference (±SD) of NF135.C10 schizonts on day three, day five, and day seven post-invasion in primary porcine and human hepatocytes based on immunofluorescence staining with HSP70. Per technical replicate, all or up to seventy-five schizonts were measured for each of the biological replicates (three human and three porcine donors). A two-way RM ANOVA was performed on the median of each replicate. **(C)** Median NF135.C10 schizont circumference on day three, day five, and day seven developing in primary porcine and human hepatocytes, as determined by immunofluorescence staining with HSP70, shown per donor. Per technical replicate, all or up to seventy-five schizonts were measured. A two-way RM ANOVA was performed. The total number of schizonts analyzed per condition is shown in **supplementary table 2**. CHH, cryopreserved human hepatocytes; FHH, fresh human hepatocytes; FPH, fresh porcine hepatocytes; HSP, heat shock protein; NS, not significant. ***P < 0.001; ****P < 0.0001.

**
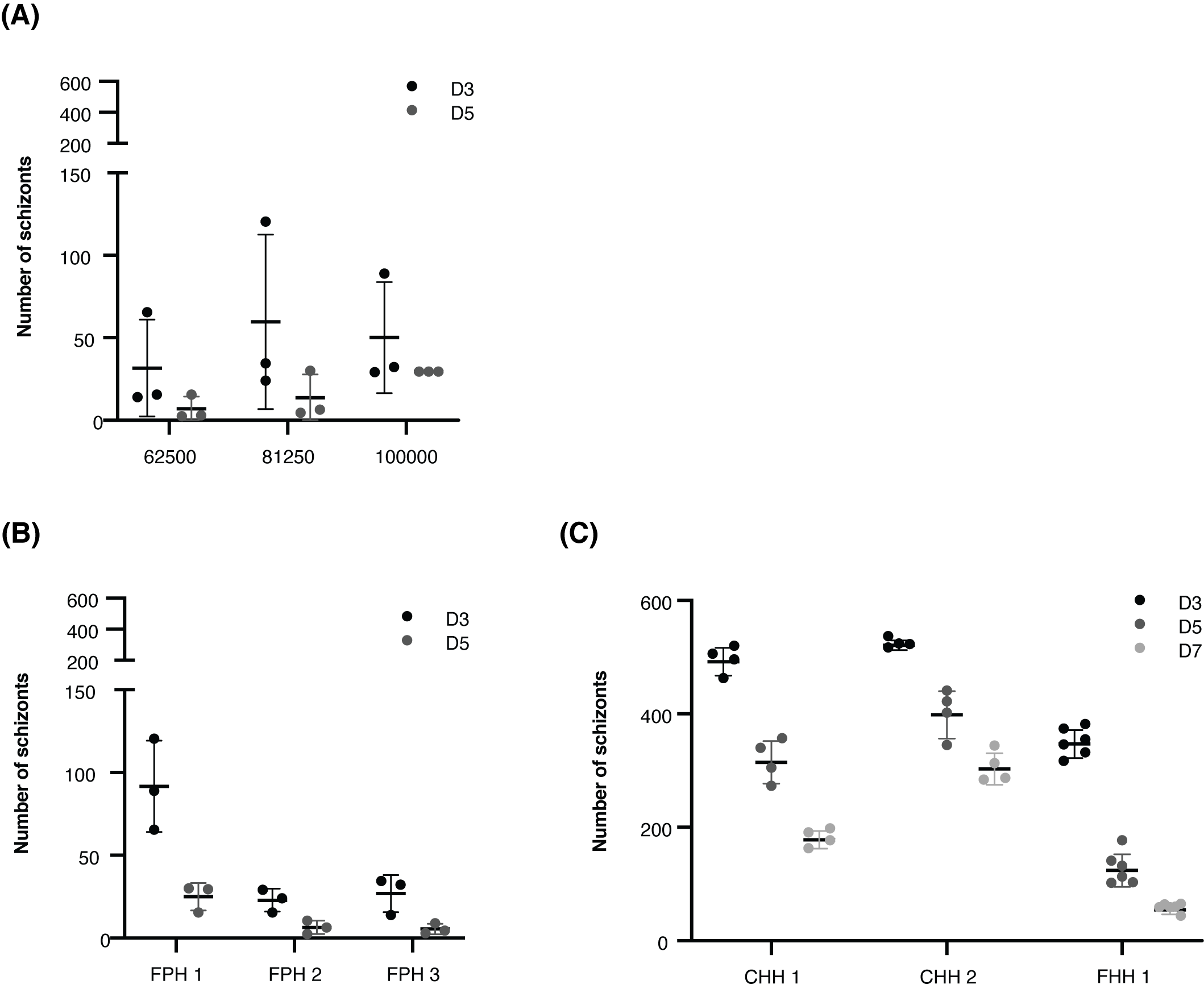
**

**Supplementary figure 2. (A)** Mean (±SD) of NF135.C10 schizonts on day three and day five post-invasion in primary porcine hepatocytes per plating density (62.500, 81.250 or 100.000 hepatocytes per well) or **(B)** per donor. Each dot represents the median of four technical replicates in three biological replicates. **(C)** Mean (±SD) of NF135.C10 schizonts on day three, day five, and day seven post-invasion in primary porcine hepatocytes per donor. Each dot represents the median of four technical replicates. CHH, cryopreserved human hepatocytes; FHH, fresh human hepatocytes; FPH, fresh porcine hepatocytes.

**
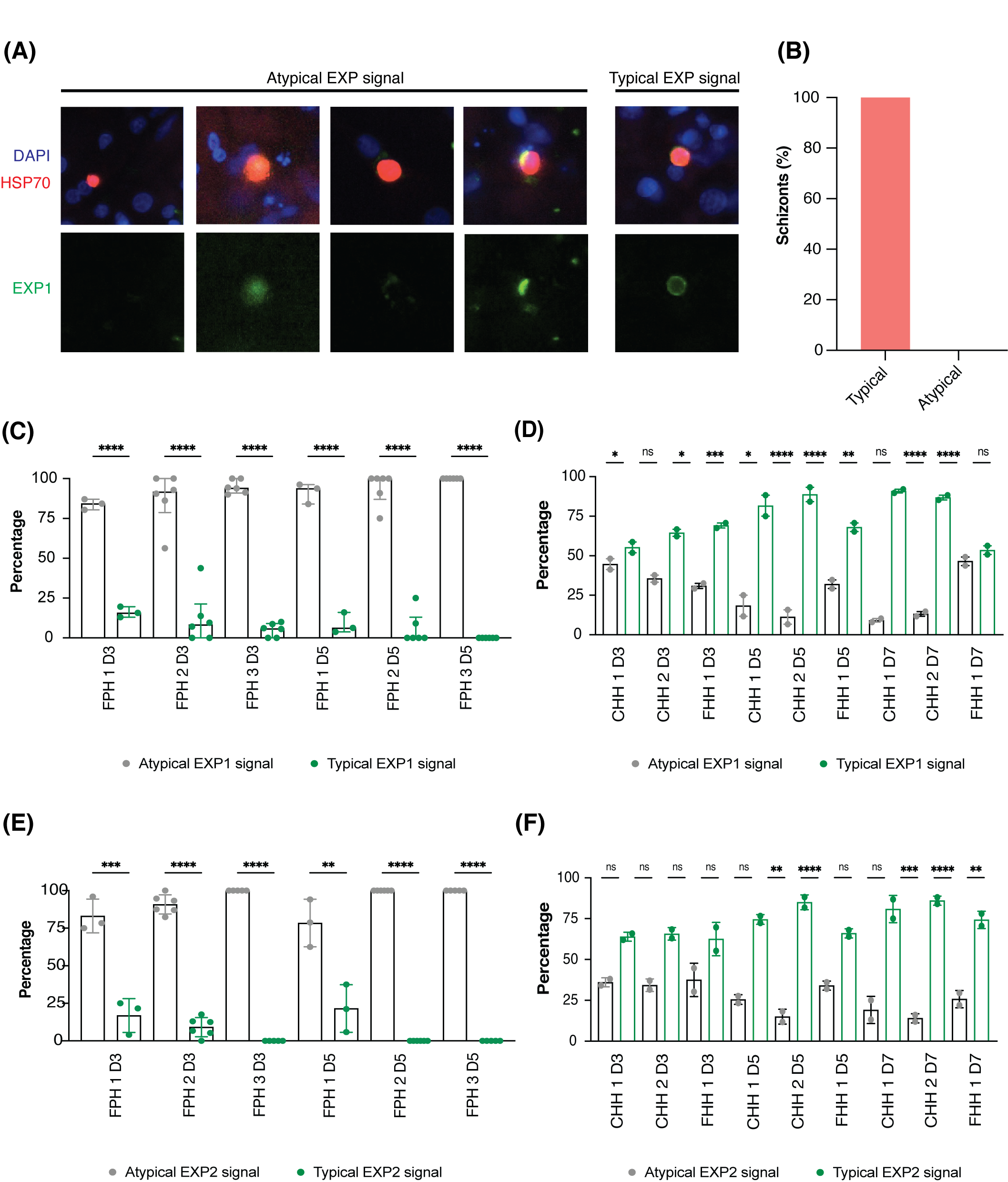
**

**Supplementary figure 3. (A)** Representative immunofluorescence images showing examples of atypical and typical EXP1 staining patterns (green). Blue: DAPI staining, red: HSP70 staining. Images were taken on the Leica High Content at 20× magnification and then cropped in Adobe Photoshop. **(B)** Mean (±SD) of the percentage of *Plasmodium berghei* schizonts in porcine hepatocytes with a typical or atypical UIS4 staining pattern. All schizonts were measured per technical replicate and a two-way RM ANOVA was performed. **(C)** Porcine hepatocytes with a typical EXP1 staining pattern. **(D)** Human hepatocytes with a typical EXP1 staining pattern. **(E)** Porcine hepatocytes with a typical EXP2 staining pattern. **(F)** Human hepatocytes with a typical EXP2 staining pattern. For figures C-F, the median (±IQR) of the percentage of schizonts in hepatocytes with a typical EXP1 or EXP2 staining pattern is shown. All or up to seventy-five schizonts were measured per technical replicate. A two-way RM ANOVA was performed. CHH, cryopreserved human hepatocytes; EXP, exported protein; FHH, fresh human hepatocytes; FPH, fresh porcine hepatocytes; HSP, heat shock protein; NS, not significant; UIS; upregulated in infectious sporozoites. *P < 0.05; **P < 0.005; ***P < 0.001; ****P < 0.0001.


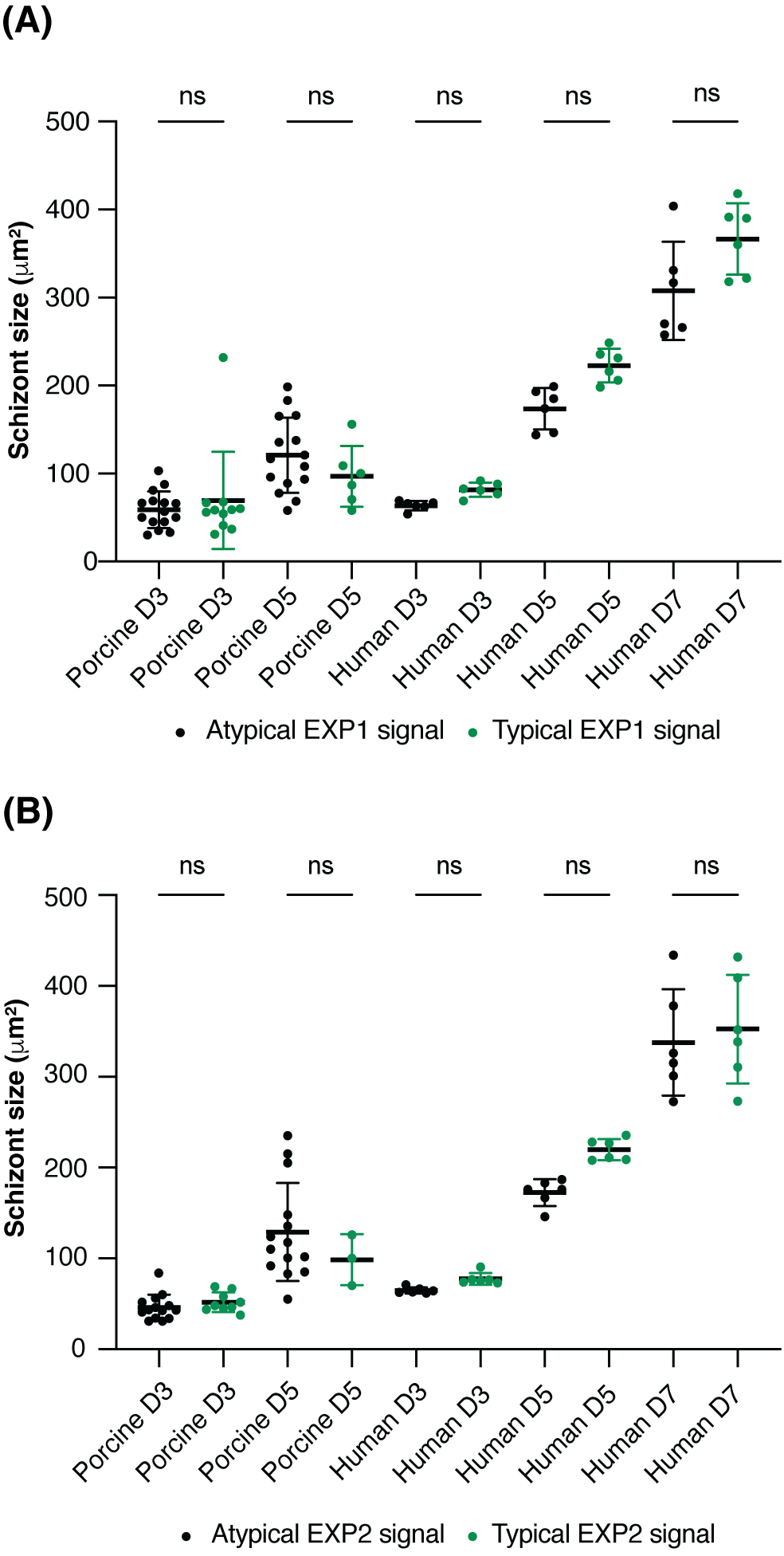


**Supplementary figure 4.** Mean (±SD) of schizont sizes schizonts developing in porcine or human hepatocytes classified as typical or atypical EXP1 **(A)** or EXP2 **(B)** staining pattern. All or up to seventy-five schizonts were measured per technical replicate. Each dot represents the median cross-sectional surface per technical replicate in three biological replicates. A two-way RM ANOVA was performed. EXP, exported protein; NS, not significant.

## Supplementary Tables

**Supplementary table 1. Schizont sizes.**

|  | **Mean area day 3 (μm^2^)**  **(SD)** | | | **Mean area day 5 (μm^2^)**  **(SD)** | | | **Mean area day 7 (μm^2^)**  **(SD)** | | |
| --- | --- | --- | --- | --- | --- | --- | --- | --- | --- |
| **Porcine** | **FPH 1** | **FPH 2** | **FPH 3** | **FPH 1** | **FPH 2** | **FPH 3** | **FPH 1** | **FPH 2** | **FPH 3** |
|  | 37.8 (15.4) | 51.2 (22.4) | 65.0 (35.2) | 114.1 (72.5) | 143.7 (108.9) | 125.3 (94.2) | N/A | NA | NA |
| **Human** | **CHH 1** | **CHH 2** | **FHH 1** | **CHH 1** | **CHH 2** | **FHH 1** | **CHH 1** | **CHH 2** | **FHH 1** |
|  | 75.7 (23.6) | 72.5 (20.3) | 77.3 (25.0) | 213.0 (76.0) | 234.4 (76.5) | 218.8 (91.3) | 429.6 (184. (3) | 386.9 (144.3) | 333.2 (136.5) |

The mean schizont area is shown for parasites developing in porcine and human hepatocytes on days three, five, and seven. The standard deviation is shown in parenthesis after each value. CHH, cryopreserved human hepatocytes; FHH, fresh human hepatocytes; FPH, fresh porcine hepatocytes; NA, not applicable; SD: standard deviation.

**Supplementary table 2. Number of schizonts measured per analysis.**

| Schizont Size | Day 3 | Day 5 | Day 7 |
| --- | --- | --- | --- |
| FPH1 | 254 | 142 | NA |
| FPH2 | 273 | 77 | NA |
| FPH3 | 304 | 69 | NA |
| CHH1 | 315 | 310 | 305 |
| CHH2 | 318 | 304 | 302 |
| FHH1 | 304 | 300 | 300 |
| HSP70 signal |  |  |  |
| Porcine | 206 | 56 | NA |
| Human | 153 | 153 | NA |
| MSP1 signal |  |  |  |
| Porcine | 206 | 56 | NA |
| Human | 153 | 153 | NA |
| EXP1 Signal – porcine hepatocytes |  |  |  |
| FPH1 | 105 | 67 | NA |
| FPH2 | 115 | 40 | NA |
| FPH3 | 129 | 37 | NA |
| EXP1 Signal – human hepatocytes |  |  |  |
| CHH1 | 154 | 154 | 153 |
| CHH2 | 161 | 152 | 152 |
| FHH1 | 152 | 150 | 123 |
| EXP2 Signal - porcine hepatocytes |  |  |  |
| FPH1 | 149 | 75 | NA |
| FPH2 | 161 | 37 | NA |
| FPH3 | 178 | 32 | NA |
| EXP2 Signal - human hepatocytes |  |  |  |
| CHH1 | 161 | 153 | 152 |
| CHH2 | 157 | 153 | 150 |
| FHH1 | 152 | 150 | 103 |

Where a maximum number of schizonts was measured per technical replicate, the total number of schizonts that were included for each analysis is shown. CHH, cryopreserved human hepatocytes; EXP, exported protein; FHH, fresh human hepatocytes; FPH: fresh porcine hepatocytes. NA: not applicable.
